# *catena*-Poly[[aqua­cadmium(II)]-bis­(μ_2_-4-chloro­benzoato)]

**DOI:** 10.1107/S1600536810008068

**Published:** 2010-03-06

**Authors:** Chong-Qing Wan, Zi-Jia Wang, Fan Zhang

**Affiliations:** a Department of Chemistry, Capital Normal University, Beijing 100048, People’s Republic of China

## Abstract

In the title complex, [Cd(C_7_H_4_ClO_2_)_2_(H_2_O)]_*n*_, the Cd atom lies on a twofold axis and adopts a square-pyramidal coordination geometry. The water mol­ecule occupies the axial site with O atoms from four different 4-chloro­benzoato ligands in the equatorial plane. Pairs of 4-chloro­benzoato ligands bridge adjacent Cd^II^ ions, generating an infinite chain structure along the *c* axis. Parallel polymeric chains are further inter­connected through water–acetate O—H⋯O hydrogen bonds, forming layers in the *bc* plane.

## Related literature

For the use of organic acids in constructing metal-organic frameworks, see: Zhao *et al.* (2003 ▶); Cao *et al.* (2002 ▶); Zhang *et al.* (2004 ▶). The related six-coordinate Cd^II^ complex with two coordinated water mol­ecules has a distorted octa­hedral geometry, see: Rodesiler *et al.* (1985 ▶). For other related structures involving the 4-chloro­benzoato anion, see: Turpeinen *et al.* (1999 ▶); Xue *et al.* (2006 ▶).
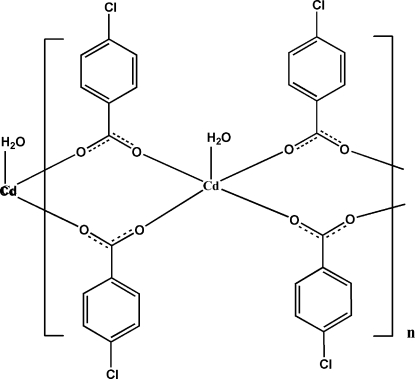


## Experimental

### 

#### Crystal data

[Cd(C_7_H_4_ClO_2_)_2_(H_2_O)]
*M* *_r_* = 441.52
Monoclinic, 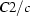

*a* = 32.525 (2) Å
*b* = 6.4769 (5) Å
*c* = 7.1419 (6) Å
β = 98.883 (3)°
*V* = 1486.48 (19) Å^3^
*Z* = 4
Mo *K*α radiation
μ = 1.85 mm^−1^
*T* = 296 K
0.4 × 0.3 × 0.2 mm

#### Data collection

Bruker APEXII CCD area-detector diffractometer
Absorption correction: multi-scan (*SADABS*; Bruker, 2007 ▶) *T*_min_ = 0.567, *T*_max_ = 0.746
9459 measured reflections
1334 independent reflections
1308 reflections with *I* > 2σ(*I*)
*R*_int_ = 0.021

#### Refinement

*R*[*F* ^2^ > 2σ(*F* ^2^)] = 0.016
*wR*(*F* ^2^) = 0.042
*S* = 1.12
1334 reflections
101 parameters
H-atom parameters constrained
Δρ_max_ = 0.26 e Å^−3^
Δρ_min_ = −0.34 e Å^−3^

### 

Data collection: *APEX2* (Bruker, 2007 ▶); cell refinement: *APEX2* and *SAINT* (Bruker, 2007 ▶); data reduction: *SAINT*; program(s) used to solve structure: *SHELXS97* (Sheldrick, 2008 ▶); program(s) used to refine structure: *SHELXL97* (Sheldrick, 2008 ▶); molecular graphics: *SHELXTL* (Sheldrick, 2008 ▶); software used to prepare material for publication: *SHELXTL* and *PLATON* (Spek, 2009 ▶).

## Supplementary Material

Crystal structure: contains datablocks I, global. DOI: 10.1107/S1600536810008068/sj2730sup1.cif

Structure factors: contains datablocks I. DOI: 10.1107/S1600536810008068/sj2730Isup2.hkl

Additional supplementary materials:  crystallographic information; 3D view; checkCIF report

## Figures and Tables

**Table 1:** Selected bond lengths (Å)

Cd1—O1^i^	2.2210 (14)
Cd1—O1*W*	2.233 (2)
Cd1—O2^ii^	2.3896 (14)

Symmetry codes: (i) 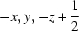; (ii) 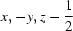.

**Table 2:** Hydrogen-bond geometry (Å, °)

*D*—H⋯*A*	*D*—H	H⋯*A*	*D*⋯*A*	*D*—H⋯*A*
O1*W*—H1*WA*⋯O2^iii^	0.89	1.88	2.699 (2)	153

Symmetry code: (iii) 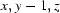.

**Fig. 1.**
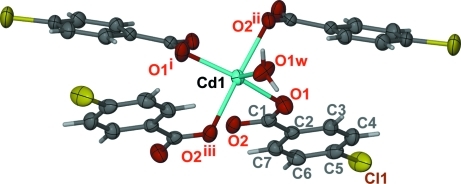
The structure of (I) showing the atom numbering scheme. Displacement ellipsoids are drawn at the 50% probability level and H atoms are shown as rods of arbitrary radius. [Symmetry Codes: (i) –x, y, –z + 1/2; (ii) –x, –y, –z + 1; (iii) x, –y, z – 1/2]

**Fig. 2.**
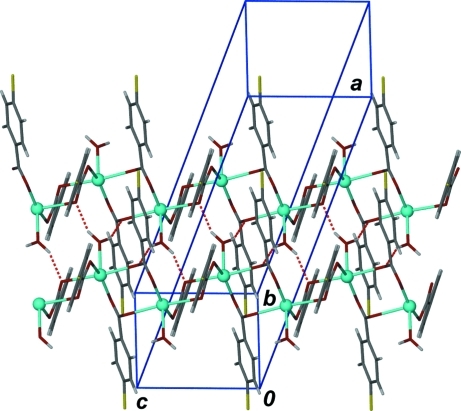
Hydrogen-bonding interactions (dashed lines) between parallel chains along the c axis of complex (I).

## supplementary crystallographic information

### Comment

Organic acids are widely used as versatile building blocks in many metal- organic frameworks with diverse structural motifs (Zhao *et al.*, 2003; Cao *et al.*, 2002; Zhang *et al.*, 2004). In metal complexes the 4-chlorobenzoato anion can function both to balance charge and as a bridging ligand. Several structures incorporating this ligand have been investigated (Turpeinen, *et al.*, 1999; Xue *et al.*, 2006).

Herein we report a new compound [Cd(C_7_H_4_ClO_2_)_2_.H_2_O]_∞_ derived from 4-chlorobenzoic acid, which exhibits an one-dimensional infinite chain structure. In the title complex the Cd^II^ atom lies on a two-fold axis and adopts a square pyramidal coordination geometry (Fig. 1). The water molecule occupies the axial site with oxygen atoms from four different 4-chlorobenzoato ligands in the equatorial plane. The Cd—O1W bond length is 2.233 (2)Å and the Cd—*O*(acetate) distances lie in the range 2.221 (1)-2.390 (1) Å, Table 1. Pairs of 4-chlorobenzoato ligands bridge two adjacent Cd^II^ ions generating an infinite chain structure along the *c* axis. Parallel polymeric chains are further interconnected through O1W—H1WA···O2 hydrogen bonds forming layers in the *bc* plane (Fig. 2, Table 2), with an O1W···O2 (D···A) distance of 2.699 (2) Å. This structure is entirely different from that of [Cd(C_7_H_4_ClO_2_)_2_.(H_2_O)_2_], in which the Cd^II^ ion adopts a distorted six-coordination geometry (Rodesiler *et al.*, 1985).

### Experimental

4-chlorobenzoic acid (0.040 g, 0.3 mmol) was dissolved in a mixture of methanol, 2 ml, and acetonitrile, 2 ml. Sodium hydroxide was subsequently added at room temperature to adjust the pH to 7. Then, Cd(ClO_4_)_2_.6H_2_O (0.371 g, 0.1 mmol) was added and the solution stirred for an hour. The clear solution was filtered and then left to stand in air. After 6 days colorless rod-like crystals were deposited (260 mg, 72% yield).

### Refinement

The hydrogen atoms were placed in idealized positions and allowed to ride on the relevant carbon atoms, with C— H = 0.93 Å and *U*ĩso~(H) = 1.2Ueq(C). The position of the hydrogen atom of the coordinated water molecule was obtained from a difference Fourier map, with the O—H distances restrained to 0.89 Å.

### Crystal data

**Table:**

[Cd(C_7_H_4_ClO_2_)_2_(H_2_O)]	*F*(000) = 864
*M**_r_* = 441.52	*D*_x_ = 1.973 Mg m^−^^3^
Monoclinic, *C*2/*c*	Mo *K*α radiation, λ = 0.71073 Å
Hall symbol: -C 2yc	Cell parameters from 543 reflections
*a* = 32.525 (2) Å	θ = 2.3–26.7°
*b* = 6.4769 (5) Å	µ = 1.85 mm^−^^1^
*c* = 7.1419 (6) Å	*T* = 296 K
β = 98.883 (3)°	Rod, colorless
*V* = 1486.48 (19) Å^3^	0.4 × 0.3 × 0.2 mm
*Z* = 4	

### Data collection

**Table:**

Bruker APEXII CCD area-detector diffractometer	1334 independent reflections
Radiation source: fine-focus sealed tube	1308 reflections with *I* > 2σ(*I*)
graphite	*R*_int_ = 0.021
ω scans	θ_max_ = 25.1°, θ_min_ = 3.2°
Absorption correction: multi-scan (*SADABS*; Bruker, 2007)	*h* = −38→38
*T*_min_ = 0.567, *T*_max_ = 0.746	*k* = −7→7
9459 measured reflections	*l* = −8→8

### Refinement

**Table:**

Refinement on *F*^2^	Primary atom site location: structure-invariant direct methods
Least-squares matrix: full	Secondary atom site location: difference Fourier map
*R*[*F*^2^ > 2σ(*F*^2^)] = 0.016	Hydrogen site location: inferred from neighbouring sites
*wR*(*F*^2^) = 0.042	H-atom parameters constrained
*S* = 1.12	*w* = 1/[σ^2^(*F*_o_^2^) + (0.0231*P*)^2^ + 1.3036*P*] *P* = (*F*_o_^2^ + 2*F*_c_^2^)/3
1334 reflections	(Δ/σ)_max_ = 0.001
101 parameters	Δρ_max_ = 0.26 e Å^−^^3^
0 restraints	Δρ_min_ = −0.34 e Å^−^^3^

### Special details

**Table:**

Geometry. All esds (except the esd in the dihedral angle between two l.s. planes) are estimated using the full covariance matrix. The cell esds are taken into account individually in the estimation of esds in distances, angles and torsion angles; correlations between esds in cell parameters are only used when they are defined by crystal symmetry. An approximate (isotropic) treatment of cell esds is used for estimating esds involving l.s. planes.
Refinement. Refinement of *F*^2^ against ALL reflections. The weighted *R*-factor wR and goodness of fit *S* are based on *F*^2^, conventional *R*-factors *R* are based on *F*, with *F* set to zero for negative *F*^2^. The threshold expression of *F*^2^ > σ(*F*^2^) is used only for calculating *R*-factors(gt) etc. and is not relevant to the choice of reflections for refinement. *R*-factors based on *F*^2^ are statistically about twice as large as those based on *F*, and *R*- factors based on ALL data will be even larger.

### Fractional atomic coordinates and isotropic or equivalent isotropic displacement parameters (Å^2^)

**Table:**

	*x*	*y*	*z*	*U*_iso_*/*U*_eq_	
Cd1	0.0000	−0.15461 (2)	0.2500	0.02841 (8)	
Cl1	0.222718 (19)	0.55886 (13)	0.63416 (9)	0.0652 (2)	
O1	0.06477 (4)	−0.0960 (2)	0.3902 (2)	0.0444 (3)	
O2	0.02952 (4)	0.1741 (2)	0.4642 (2)	0.0361 (3)	
C1	0.06390 (6)	0.0837 (3)	0.4530 (2)	0.0306 (4)	
C2	0.10373 (6)	0.1976 (3)	0.5081 (2)	0.0298 (4)	
C3	0.10333 (6)	0.4005 (3)	0.5697 (3)	0.0355 (4)	
H3A	0.0782	0.4630	0.5836	0.043*	
C4	0.14008 (6)	0.5103 (3)	0.6107 (3)	0.0405 (5)	
H4A	0.1398	0.6468	0.6510	0.049*	
C5	0.17716 (6)	0.4147 (4)	0.5911 (3)	0.0407 (5)	
C6	0.17853 (6)	0.2115 (4)	0.5343 (3)	0.0421 (5)	
H6A	0.2038	0.1485	0.5249	0.050*	
C7	0.14156 (6)	0.1033 (3)	0.4914 (3)	0.0363 (4)	
H7A	0.1420	−0.0332	0.4513	0.044*	
O1W	0.0000	−0.4993 (3)	0.2500	0.0527 (6)	
H1WA	0.0106	−0.5756	0.3494	0.079*	

### Atomic displacement parameters (Å^2^)

**Table:**

	*U*^11^	*U*^22^	*U*^33^	*U*^12^	*U*^13^	*U*^23^
Cd1	0.02668 (12)	0.02429 (12)	0.03493 (12)	0.000	0.00687 (8)	0.000
Cl1	0.0426 (3)	0.0929 (5)	0.0609 (4)	−0.0347 (3)	0.0102 (3)	−0.0183 (3)
O1	0.0365 (8)	0.0428 (8)	0.0546 (9)	−0.0097 (6)	0.0093 (6)	−0.0179 (7)
O2	0.0272 (7)	0.0388 (7)	0.0420 (8)	−0.0023 (5)	0.0047 (6)	0.0094 (5)
C1	0.0300 (9)	0.0369 (10)	0.0252 (8)	−0.0059 (8)	0.0047 (7)	0.0034 (7)
C2	0.0282 (9)	0.0356 (9)	0.0260 (8)	−0.0046 (7)	0.0047 (7)	0.0006 (7)
C3	0.0316 (10)	0.0396 (10)	0.0360 (9)	−0.0026 (8)	0.0075 (8)	−0.0046 (8)
C4	0.0434 (11)	0.0411 (11)	0.0377 (10)	−0.0117 (9)	0.0081 (8)	−0.0080 (8)
C5	0.0323 (10)	0.0579 (13)	0.0315 (9)	−0.0160 (9)	0.0036 (8)	−0.0014 (9)
C6	0.0273 (10)	0.0556 (12)	0.0434 (11)	0.0006 (9)	0.0055 (8)	0.0032 (10)
C7	0.0328 (10)	0.0374 (10)	0.0390 (10)	0.0000 (8)	0.0069 (8)	0.0016 (8)
O1W	0.0701 (15)	0.0225 (10)	0.0564 (13)	0.000	−0.0190 (11)	0.000

### Geometric parameters (Å, °)

**Table:**

Cd1—O1^i^	2.2210 (14)	C2—C7	1.395 (3)
Cd1—O1	2.2210 (14)	C3—C4	1.383 (3)
Cd1—O1W	2.233 (2)	C3—H3A	0.9300
Cd1—O2^ii^	2.3896 (14)	C4—C5	1.382 (3)
Cd1—O2^iii^	2.3896 (14)	C4—H4A	0.9300
Cl1—C5	1.738 (2)	C5—C6	1.380 (3)
O1—C1	1.249 (2)	C6—C7	1.385 (3)
O2—C1	1.276 (2)	C6—H6A	0.9300
O2—Cd1^iii^	2.3896 (14)	C7—H7A	0.9300
C1—C2	1.490 (3)	O1W—H1WA	0.8900
C2—C3	1.386 (3)		
			
O1^i^—Cd1—O1	160.33 (8)	C7—C2—C1	120.18 (18)
O1^i^—Cd1—O1W	99.84 (4)	C4—C3—C2	120.30 (18)
O1—Cd1—O1W	99.84 (4)	C4—C3—H3A	119.9
O1^i^—Cd1—O2^ii^	95.88 (5)	C2—C3—H3A	119.9
O1—Cd1—O2^ii^	85.16 (5)	C5—C4—C3	119.15 (19)
O1W—Cd1—O2^ii^	86.97 (3)	C5—C4—H4A	120.4
O1^i^—Cd1—O2^iii^	85.16 (5)	C3—C4—H4A	120.4
O1—Cd1—O2^iii^	95.88 (5)	C6—C5—C4	121.72 (18)
O1W—Cd1—O2^iii^	86.97 (3)	C6—C5—Cl1	119.92 (16)
O2^ii^—Cd1—O2^iii^	173.94 (6)	C4—C5—Cl1	118.34 (17)
C1—O1—Cd1	104.39 (12)	C5—C6—C7	118.77 (19)
C1—O2—Cd1^iii^	120.07 (11)	C5—C6—H6A	120.6
O1—C1—O2	121.26 (17)	C7—C6—H6A	120.6
O1—C1—C2	119.30 (17)	C6—C7—C2	120.4 (2)
O2—C1—C2	119.39 (17)	C6—C7—H7A	119.8
C3—C2—C7	119.59 (18)	C2—C7—H7A	119.8
C3—C2—C1	120.18 (17)	Cd1—O1W—H1WA	123.7
			
O1^i^—Cd1—O1—C1	21.30 (11)	O2—C1—C2—C7	−178.09 (17)
O1W—Cd1—O1—C1	−158.70 (11)	C7—C2—C3—C4	1.4 (3)
O2^ii^—Cd1—O1—C1	115.23 (12)	C1—C2—C3—C4	−176.33 (18)
O2^iii^—Cd1—O1—C1	−70.77 (12)	C2—C3—C4—C5	−0.6 (3)
Cd1—O1—C1—O2	14.4 (2)	C3—C4—C5—C6	−0.9 (3)
Cd1—O1—C1—C2	−162.92 (13)	C3—C4—C5—Cl1	177.43 (16)
Cd1^iii^—O2—C1—O1	92.47 (18)	C4—C5—C6—C7	1.6 (3)
Cd1^iii^—O2—C1—C2	−90.19 (17)	Cl1—C5—C6—C7	−176.69 (16)
O1—C1—C2—C3	176.98 (18)	C5—C6—C7—C2	−0.8 (3)
O2—C1—C2—C3	−0.4 (3)	C3—C2—C7—C6	−0.6 (3)
O1—C1—C2—C7	−0.7 (3)	C1—C2—C7—C6	177.07 (18)

Symmetry codes: (i) −*x*, *y*, −*z*+1/2; (ii) *x*, −*y*, *z*−1/2; (iii) −*x*, −*y*, −*z*+1.

### Hydrogen-bond geometry (Å, °)

**Table:**

*D*—H···*A*	*D*—H	H···*A*	*D*···*A*	*D*—H···*A*
O1W—H1WA···O2^iv^	0.89	1.88	2.699 (2)	153

Symmetry codes: (iv) *x*, *y*−1, *z*.
